# Associations Between Sarcopenia, Heart Failure and Myocardial Infarction in Patients With Systemic Lupus Erythematosus and Rheumatoid Arthritis

**DOI:** 10.3389/fmed.2022.882911

**Published:** 2022-06-28

**Authors:** Ching-Mao Chang, Jr-Rung Lin, Tieh-Cheng Fu

**Affiliations:** ^1^Center for Traditional Medicine, Taipei Veterans General Hospital, Taipei, Taiwan; ^2^Faculty of Medicine, National Yang Ming Chiao Tung University, Taipei, Taiwan; ^3^School of Medicine, Institute of Traditional Medicine, National Yang Ming Chiao Tung University, Taipei, Taiwan; ^4^Clinical Informatics and Medical Statistics Research Center, Graduate Institute of Clinical Medical (Joint Appointment), Chang Gung University, Taoyuan, Taiwan; ^5^Department of Physical Medicine and Rehabilitation, Chang Gung Memorial Hospital, Keelung, Taiwan; ^6^Division of Cardiology, Department of Internal Medicine, Heart Failure Research Center, Chang Gung Memorial Hospital, Keelung, Taiwan; ^7^School of Medicine, College of Medicine, Chang Gung University, Taoyuan, Taiwan

**Keywords:** sarcopenia, heart failure, rheumatoid arthritis, SLE, myocardial infarction

## Abstract

**Objectives:**

To evaluate associations between sarcopenia, type of autoimmune disease and risk of heart failure (HF) and myocardial infarction (MI) in patients with systemic lupus erythematosus (SLE) and rheumatoid arthritis (RA).

**Methods:**

In this population-based, cross-sectional study, discharge data from the 2005–2014 US Nationwide Inpatient Sample (NIS) of hospitalized patients with SLE or RA were extracted and analyzed. Univariate and multivariable regression analyses were conducted to determine associations between sarcopenia, type of autoimmune disease and risk of HF/MI.

**Results:**

After exclusions, 781,199 hospitalized patients diagnosed with SLE or RA were included. Among the study cohort, 127,812 (16.4%) were hospitalized with HF, and 12,781 (1.6%) were hospitalized with MI. Sarcopenia was found in only 0.1% of HF/MI patients. Logistic regression analyses revealed that sarcopenia was not significantly associated with presence of either HF or MI. Patients with RA had significantly lower odds of HF than SLE patients (aOR = 0.77, 95%CI: 0.76, 0.79) or MI (aOR = 0.86, 95%CI: 0.82, 0.91).

**Conclusion:**

In the US, among hospitalized adults diagnosed with SLE or RA, patients with RA are significantly less likely to have HF or MI than those with SLE. Whether sarcopenia leads to increased HF or MI remains inconclusive. Further studies are warranted to investigate the pathophysiology underlying discrepancies between RA and SLE regarding risk for MI or HF.

## Introduction

Systemic lupus erythematosus (SLE) and rheumatoid arthritis (RA) are relatively common autoimmune disorders in which the immune system selectively attacks certain healthy tissue in the body, affecting joints and muscles and causing persistent systemic inflammation, pain, reduced physical activity, extra-articular complications, loss of muscle mass and muscle strength, and altered body composition; together these complications lead to daily functional disability and reduced quality of life (1–4). Increased risk of cardiovascular diseases such as heart failure (HF), acute myocardial infarction (AMI), or atherosclerotic cardiovascular disease (ASCVD) were observed in patients with SLE (5–10). In addition, previous studies have shown that an excess burden of CVD in patients with RA is an established pattern (11, 12). However, no prior study directly compared the risk of HF or MI between patients with SLE vs. those with RA.

Previous studies on body composition have reported that sarcopenia is highly prevalent in HF patients, suggesting that it contributes to poor prognosis, and that age-related muscle decline may be the most critical contributor to reduced cardiorespiratory fitness in older adults with HF (13–15). Importantly, systemic inflammation may represent a risk for altered body composition. Women with SLE or RA are more likely to have an abnormal body composition phenotype, although differences exist between the two diseases (16). Other studies have also reported a higher prevalence of sarcopenia and sarcopenic obesity in RA patients (17, 18). However, more evidence is needed before consensus can be reached on the associations between sarcopenia, SLE and RA. Therefore, the present study aimed to evaluate the associations between sarcopenia, HF and MI and the relative risk of these diseases in patients with autoimmune SLE and RA.

## Materials and Methods

### Study Design and Data Source

This population-based, cross-sectional study extracted data from the US Nationwide Inpatient Sample (NIS) database, which is the largest all-payer, inpatient care database in the US, including about 8 million hospital stays each year (19). The database is administered by the Healthcare Cost and Utilization Project (HCUP) of the US National Institutes of Health (NIH) and developed through a Federal-State-Industry partnership and sponsored by the Agency for Healthcare Research and Quality (AHRQ). All admitted patients are initially considered for inclusion. The continuous, annually updated NIS database derives patient data of about 1,050 hospitals from 44 States in the US, representing a 20% stratified sample of the US community hospitals as defined by the American Hospital Association.

### Ethics Statement

All data were obtained through request to the Online HCUP Central Distributor (available at: https://www.distributor.hcup-us.ahrq.gov/), which administers the database (certificate # HCUP-833FWV78H). This study conforms to the NIS data-use agreement with HCUP. As this study analyzed secondary data of the NIS database, patients' data were not used directly used. This study was approved by the Institutional Review Board of the Chang Gung Medical Hospital (202100234B0). As we conducted this retrospective analysis of the NHIRD and all the individual information were de-identified, we could not obtain informed consent from the recruited patients.

### Study Population

Adults ≥20 years old admitted to US hospitals between 2005 and 2014 with a primary or secondary diagnosis of SLE or RA were identified in the NIS database through the International Classification of Diseases, Ninth Revision (ICD-9) diagnostic codes (code 710.0 or 714.0). Patients with congenital heart disease, asthma, HIV, heart surgery, and cancer were excluded. Patients with incomplete data for outcomes and main variables of interest were also excluded from the study cohort.

### HF, MI, and Sarcopenia

HF was identified by ICD-9 code 428.x, which covered any type of HF regardless of ejection or reduced ejection fractions. MI was identified through code 410.x, which covered all types of MI. Since no unique code for “sarcopenia” is included in the ICD-9 coding system, sarcopenia was defined using the code 728.2x (muscular wasting and disuse atrophy) in accordance with a previous study (20).

### Covariates

Patients' baseline characteristics included age, gender, race (ethnicity), household income level, insurance status (primary payer), admission type, smoking, alcohol consumption, diabetes, hypertension, and hyperlipidemia. In addition, hospital characteristics, including hospital bed size, location, hospital region, and length of stay, were also extracted from the database as part of the comprehensive data available for all patients in the NIS database. Length of stay is calculated by subtracting the admission date from the discharge date. Details on hospital characteristics are documented on the NIS webpage (https://www.hcup-us.ahrq.gov/db/vars/hosp_locteach/nisnote.jsp).

### Statistical Analysis

Descriptive statistics of the patients are presented as unweighted counts (n) and weighted percentage (%) or mean ± standard error (SE). Differences between the groups were evaluated using PROC SURVEYFREQ and SURVEYREG for analysis of categorical and continuous data. Logistic regression analysis was performed using PROC SURVEYLOGISTIC to determine factors associated with the presence of HF and MI. Variables that were significant in univariate regression analysis were adjusted in multivariable analysis. Since the NIS database covers 20% of samples of the USA annual inpatient admissions, weighted samples (DISCWT), stratum (NIS_STRATUM), cluster (HOSPID) were used to generate national estimates for all analyses. All *p-*values were two-sided and *p* < 0.05 was considered statistically significant. All statistical analyses were carried out with SAS 9.4. A two-tailed *P*-value < 0.05 was considered statistically significant.

## Results

### Patient Selection and Study Cohort

**Figure 1:**
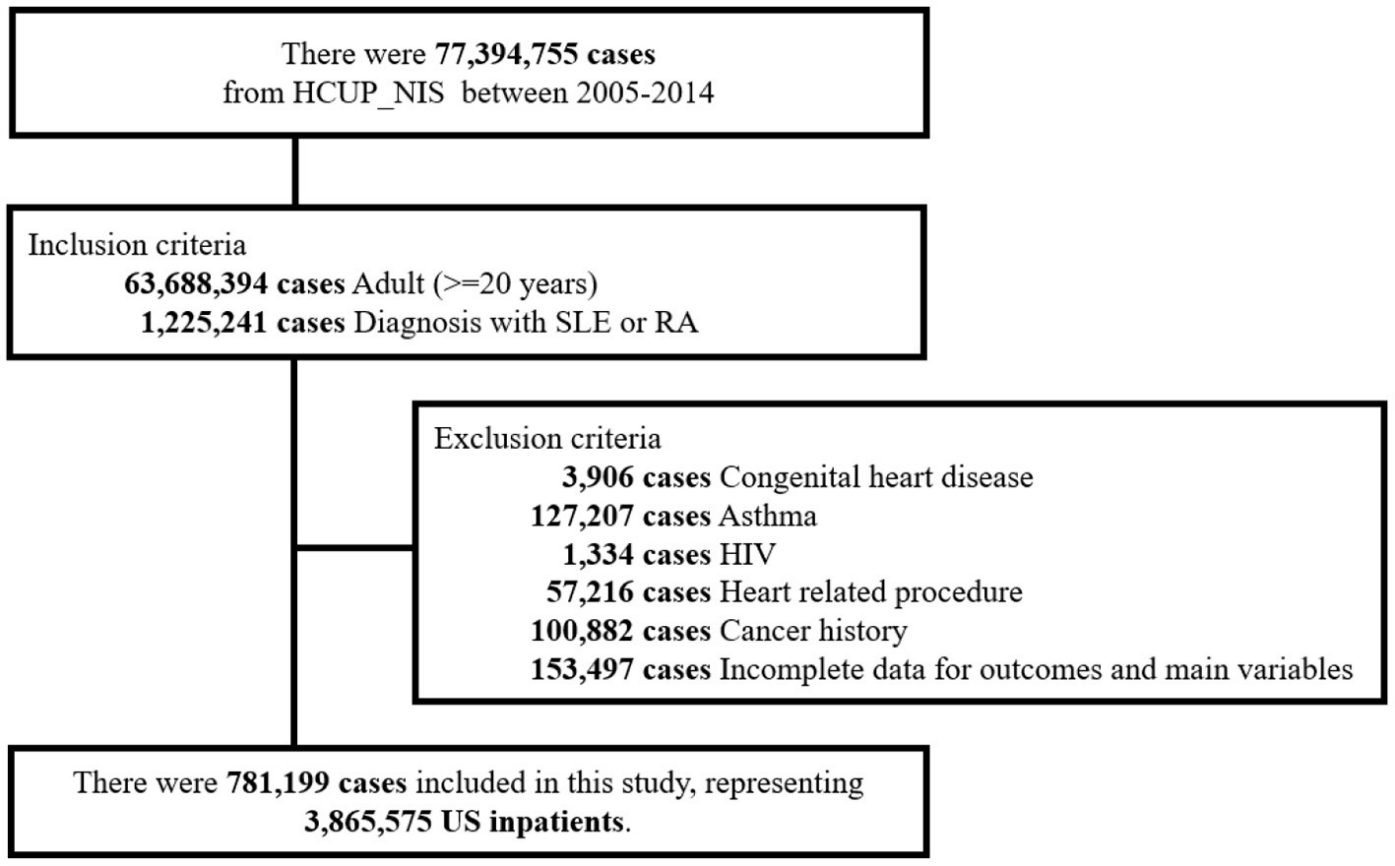
Flow diagram of study population.

The detailed patient selection process is shown in Figure 1. Data of 1,225,241 patients aged 20 years or older who were diagnosed with SLE, RA or both were extracted. After excluding patients with congenital heart diseases (*n* = 3,906), asthma (*n* = 127,207), HIV (*n* = 1,334), previous cardiac procedure (*n* = 57,216), cancer history (*n* = 100,882), and incomplete data (*n* = 153,497), finally 781,199 patients (representing 3,865,575 US inpatients) were included as the study cohort (Figure 1).

A total of 0.1% patients had sarcopenia, 25.6% had SLE alone, 71.2% had RA alone, and 3.2% had overlapping diagnoses between SLE and RA. Mean age was 63.2, and the majority of patients were female (79.1%), White (71.0%), non-smokers (99.9%) and did not consume alcohol (99.6%). Most patients were admitted emergently (77.8%), with income levels at the lowest quartile (29.6%), and insurance covered by Medicare/Medicaid (72.6%) (Table 1).

**Table 1:** Characteristics of SLE/RA patients with or without HF.

**Study variables**	**Total**	**HF**		
	**(*n =* 781,199)**	**Yes**	**No**	***P*-value**
		**(*n =* 127,812)**	**(*n =* 653,387)**	
**Sarcopenia**				**0.003**
No	780,405 (99.9)	127,651 (99.9)	652,754 (99.9)	
Yes	794 (0.1)	161 (0.1)	633 (0.1)	
**Autoimmune disease**				**<0.001**
SLE alone	200,007 (25.6)	27,529 (21.5)	172,478 (26.4)	
RA alone	555,837 (71.2)	96,886 (75.9)	458,951 (70.3)	
Both	25,355 (3.2)	3,397 (2.7)	21,958 (3.4)	
**Patients' characteristics**				
**Age, years**	63.2 ± 0.07	71.4 ± 0.08	61.7 ± 0.07	**<0.001**
20 to <45	121,423 (15.5)	7,259 (5.6)	114,164 (17.4)	**<0.001**
45–64	254,411 (32.5)	27,532 (21.5)	226,879 (34.7)	
65+	405,365 (52.0)	93,021 (72.8)	312,344 (47.9)	
**Gender**				**<0.001**
Male	163,205 (20.9)	31,480 (24.7)	131,725 (20.2)	
Female	617,994 (79.1)	96,332 (75.3)	521,662 (79.8)	
**Race/ethnicity**				**<0.001**
White	554,326 (71.0)	91,715 (71.8)	462,611 (70.8)	
Black	124,166 (15.9)	23,240 (18.2)	100,926 (15.4)	
Hispanic	67,856 (8.7)	8,086 (6.3)	59,770 (9.1)	
Asian/Pacific Islander	12,497 (1.6)	1,659 (1.3)	10,838 (1.6)	
Native American	5,653 (0.7)	772 (0.6)	4,881 (0.8)	
Others	16,701 (2.2)	2340 (1.8)	14,361 (2.2)	
**Household Income**				**<0.001**
Lowest quartile	230,541 (29.6)	40,692 (31.9)	189,849 (29.1)	
Second quartile	200,960 (25.7)	33,224 (26.0)	167,736 (25.7)	
Third quartile	185,559 (23.7)	29,167 (22.8)	156,392 (23.9)	
Fourth quartile	164,139 (21.0)	24,729 (19.3)	139,410 (21.3)	
**Insurance status/Primary Payer**				**<0.001**
Medicare/Medicaid	566,518 (72.6)	111,201 (87.0)	455,317 (69.7)	
Private including HMO	176,900 (22.6)	13,473 (10.5)	163,427 (25.0)	
Self-pay/no charge/other	37,781 (4.8)	3,138 (2.5)	34,643 (5.3)	
**Admission type**				**<0.001**
Elective	174,045 (22.2)	14,086 (11.0)	159,959 (24.4)	
Emergent	607,154 (77.8)	113,726 (89.0)	493,428 (75.6)	
**Smoking**				0.149
No	780,109 (99.9)	127,652 (99.9)	652,457 (99.9)	
Yes	1,090 (0.1)	160 (0.1)	930 (0.1)	
**Alcohol consumption**				**<0.001**
No	777,879 (99.6)	127,585 (99.8)	650,294 (99.5)	
Yes	3,320 (0.4)	227 (0.2)	3,093 (0.5)	
**Diabetes**				**<0.001**
No	602,763 (77.1)	85,242 (66.7)	517,521 (79.2)	
Yes	178,436 (22.9)	42,570 (33.3)	135,866 (20.8)	
**Hypertension**				**<0.001**
No	773,213 (99.0)	124,801 (97.7)	648,412 (99.2)	
Yes	7,986 (1.0)	3,011 (2.3)	4,975 (0.8)	
**Hyperlipidemia**				**<0.001**
No	575,079 (73.6)	86,219 (67.4)	488,860 (74.8)	
Yes	206,120 (26.4)	41,593 (32.6)	164,527 (25.2)	
**Hospital characteristics**				
**Hospital bed size**				**<0.001**
Large	468,746 (60.2)	74,911 (58.8)	393,835 (60.5)	
Medium	199,574 (25.6)	33,590 (26.3)	165,984 (25.5)	
Small	112,879 (14.2)	19,311 (14.9)	93,568 (14.0)	
**Location/teaching status**				**<0.001**
Rural	92,880 (12.0)	17,963 (14.2)	74917 (11.6)	
Urban-nonteaching	334,551 (42.6)	54,396 (42.3)	280,155 (42.6)	
Urban-teaching	353,768 (45.4)	55,453 (43.5)	298,315 (45.8)	
**Hospital region**				**<0.001**
Northeast	152,063 (19.9)	24,267 (19.4)	127,796 (20.0)	
Midwest	141,999 (18.3)	26,491 (20.9)	115508 (17.8)	
South	335,202 (42.7)	54,740 (42.6)	280462 (42.7)	
West	151,935 (19.1)	22,314 (17.1)	129,621 (19.5)	
**Length of stay, days**	5.1 ± 0.01	6.2 ± 0.03	4.9 ± 0.01	**<0.001**

*HF, heart failure; HMO, Health maintenance organization; RA: rheumatoid arthritis; SE, standard error; SLE, systemic lupus erythematosus. Categorical data are presented as unweighted counts (weighted %); continuous data are presented as mean ± SE. Significant results were shown in bold*.

### Characteristic of Patients With or Without HF

Characteristics of patients with or without HF are summarized in Table 1. Among all patients, 127,812 (16.4%) had HF and 653,387 (83.6%) did not. Patients with HF had higher proportions of sarcopenia (0.13% vs. 0.10%, *p*-value = 0.003) and RA (75.9% vs. 70.3%, *p* < 0.001) than those without HF. Also, HF patients were older (74.1 vs. 61.7 years, *p* < 0.001), stayed longer in the hospital (6.2 vs. 4.9 days, *p*-value < 0.001), included more males (24.7% vs. 20.2%), were White (71.8% vs. 70.8%) or Black race (18.2% vs. 15.4%), had lower household income (Q1:31.9% vs. 29.1%; Q2: 26.0% vs. 25.7%), insurance covered by Medicare/Medicaid (87.0% vs. 69.7%), emergent admission (89.0% vs. 75.6%), were non-drinkers (99.8% vs. 99.5%) and had more diabetes, hypertension and hyperlipidemia (all *p*-value < 0.001) (Table 1).

### Characteristics of Patients With or Without MI

Characteristics of patients with or without MI are summarized in Table 2. Among all patients, 12,781 (1.6%) had MI, and 768,418 (98.4%) did not. RA occurred more frequently in MI patients (79.6% vs. 71.1%, *p*-value < 0.001) than in those without MI. The incidence of sarcopenia was not significantly different between patients with or without MI (Table 2).

**Table 2:** Characteristics of SLE/RA patients with or without MI.

**Study variables**	**MI**		
	**Yes**	**No**	***P*-value**
	**(*n =* 12,781)**	**(*n =* 768,418)**	
**Sarcopenia**			0.568
No	12,770 (99.9)	767,635 (99.9)	
Yes	11 (0.1)	783 (0.1)	
**Autoimmune disease**			**<0.001**
SLE alone	2,323 (18.2)	197,684 (25.7)	
RA alone	10,175 (79.6)	545,662 (71.1)	
Both	283 (2.2)	25,072 (3.3)	
**Patients' characteristics**			
**Age, years**	73.4 ± 0.13	63.1 ± 0.07	**<0.001**
20 to <45	421 (3.3)	121,002 (15.7)	**<0.001**
45–64	2,498 (19.5)	251,913 (32.8)	
65+	9,862 (77.2)	395,503 (51.5)	
**Gender**			**<0.001**
Male	3,376 (26.4)	159,829 (20.8)	
Female	9,405 (73.6)	608,589 (79.2)	
**Race/ethnicity**			**<0.001**
White	9,949 (77.9)	544,377 (70.9)	
Black	1,479 (11.6)	122,687 (16.0)	
Hispanic	815 (6.3)	67,041 (8.7)	
Asian/Pacific Islander	220 (1.7)	12,277 (1.6)	
Native American	77 (0.6)	5,576 (0.7)	
Others	241 (1.9)	16,460 (2.2)	
**Household Income**			**0.044**
Lowest Quartile	3,607 (28.3)	226,934 (29.6)	
Second quartile	3,383 (26.5)	197,577 (25.7)	
Third quartile	3,049 (23.8)	182,510 (23.7)	
Fourth quartile	2,742 (21.4)	161,397 (21.0)	
**Insurance status/Primary Payer**			**<0.001**
Medicare/Medicaid	10,921 (85.5)	555,597 (72.3)	
Private including HMO	1,511 (11.8)	175,389 (22.8)	
Self-pay/no charge/other	349 (2.7)	37,432 (4.9)	
**Admission type**			**<0.001**
Elective	972 (7.6)	173,073 (22.5)	
Emergent	11,809 (92.4)	595,345 (77.5)	
**Smoking**			0.817
No	12,764 (99.9)	767,345 (99.9)	
Yes	17 (0.1)	1,073 (0.1)	
**Alcohol consumption**			0.413
No	12,733 (99.6)	765,146 (99.6)	
Yes	48 (0.4)	3,272 (0.4)	
**Diabetes**			**<0.001**
No	9,079 (71.0)	593,684 (77.2)	
Yes	3,702 (29.0)	174,734 (22.8)	
**Hypertension**			**<0.001**
No	12,552 (98.2)	760,661 (99.0)	
Yes	229 (1.8)	7,757 (1.0)	
**Hyperlipidemia**			**<0.001**
No	8,047 (62.9)	567,032 (73.7)	
Yes	4,734 (37.1)	201,386 (26.3)	
**Hospital characteristics**			
**Hospital bed size**			**<0.001**
Large	7,262 (57.1)	461,484 (60.2)	
Medium	3,466 (27.1)	196,108 (25.6)	
Small	2,053 (15.8)	110,826 (14.2)	
**Location/teaching status**			**<0.001**
Rural	1,881 (14.9)	90,999 (11.9)	
Urban-nonteaching	5,666 (44.0)	328,885 (42.6)	
Urban-teaching	5,234 (41.0)	348,534 (45.5)	
**Hospital region**			**<0.001**
Northeast	3,009 (24.1)	149,054 (19.8)	
Midwest	2,402 (18.9)	139,597 (18.3)	
South	4,876 (37.9)	330,326 (42.8)	
West	2,494 (19.1)	149,441 (19.1)	
**Length of stay, days**	6.6 ± 0.07	5.1 ± 0.01	**<0.001**

*HMO, Health maintenance organization; MI, myocardial infarction; RA, rheumatoid arthritis; SE, standard error; SLE, systemic lupus erythematosus. Categorical data are presented as unweighted counts (weighted %); continuous data are presented as mean ± SE. Significant results were shown in bold*.

### Prevalence of Sarcopenia in Patients With HF or MI, Stratified by SLE/RA Diagnosis

As shown in Table 3, prevalence of sarcopenia among HF patients was 0.1, 0.1, and 0.2% among patients diagnosed with SLE alone, RA alone, and both SLE and RA, respectively. In addition, prevalence of sarcopenia among MI patients was 0.1, 0.1 and 0% among patients diagnosed with SLE alone, RA alone and both, respectively.

**Table 3:** Prevalence of sarcopenia in patients with HF or MI, stratified by SLE/RA.

	**Total**	**Autoimmune disease**			***P*-value**
		**SLE alone**	**RA alone**	**Both**	
**HF**					0.019
No sarcopenia	127,651 (99.9)	27,507 (99.9)	96,755 (99.9)	3,389 (99.8)	
Sarcopenia	161 (0.1)	22 (0.1)	131 (0.1)	8 (0.2)	
**MI**					–
No sarcopenia	12,770 (99.9)	2,321 (99.9)	10,166 (99.9)	283 (100.0)	
Sarcopenia	11 (0.1)	2 (0.1)	9 (0.1)	-	

*HF, heart failure; MI, myocardial infarction; RA, rheumatoid arthritis; SLE, systemic lupus erythematosus*.

### Associations Between Sarcopenia, Type of Autoimmune Disease and Presence of HF

The results of univariate and multivariate regression analysis on the associations between sarcopenia, type of autoimmune disease and HF are summarized in Table 4. After adjusting for relevant confounders, no significant associations were found between sarcopenia and odds for HF (aOR: 1.12, 95% CI: 0.94–1.33). Patients with RA had significantly lower odds of HF occurrence (aOR = 0.77, 95% CI: 0.76–0.79) than SLE patients.

**Table 4:** Associations between sarcopenia, type of autoimmune disease and the presence of HF.

	**Univariate**		**Multivariate**	
	**OR (95% CI)**	***p*-value**	**aOR (95% CI)**	***p*-value**
**Sarcopenia**				
No	Ref	Ref	Ref	Ref
Yes	**1.30 (1.09, 1.54)**	**0.003**	1.12 (0.94, 1.33)	0.219
**Autoimmune disease**				
SLE	Ref	Ref	Ref	Ref
RA	**1.32 (1.30, 1.35)**	**<0.001**	**0.77 (0.76, 0.79)**	**<0.001**

*CI, confidence interval; aOR, adjusted odds ratio; HF, heart failure; RA, rheumatoid arthritis; SLE, systemic lupus erythematosus. Adjusted for age, gender, race/ethnicity, household income, insurance status/primary payer, admission type, alcohol consumption, diabetes, hypertension, hyperlipidemia, hospital bed size, location/teaching status, hospital region. Significant results were shown in bold*.

### Associations Between Sarcopenia, Type of Autoimmune Disease and the Presence of MI

The results of univariate and multivariate regression analysis on associations between sarcopenia, type of autoimmune disease and MI are summarized in Table 5. Univariate analysis revealed no associations between sarcopenia and odds for MI. After adjusting for relevant confounders, RA patients had significantly lower odds for MI (aOR = 0.86, 95% CI: 0.82–0.91) than SLE patients.

**Table 5:** Associations between sarcopenia, type of autoimmune disease and the presence of MI.

	**Univariate**		**Multivariate**	
	**OR (95% CI)**	***p*-value**	**aOR (95% CI)**	***p*-value**
**Sarcopenia**				
No	Ref	Ref	Ref	Ref
Yes	0.82 (0.41, 1.63)	0.569	–	–
**Autoimmune disease**				
SLE	Ref	Ref	Ref	Ref
RA	**1.58 (1.51, 1.66)**	**<0.001**	**0.86 (0.82, 0.91)**	**<0.001**

*CI, confidence interval; aOR, adjusted odds ratio; MI, myocardial infarction; RA, rheumatoid arthritis; SLE, systemic lupus erythematosus. Adjusted for age, gender, race/ethnicity, household income, insurance status / primary payer, admission type, diabetes, hypertension, hyperlopidemia, hospital bed size, location/teaching status, hospital region. Significant results were shown in bold*.

## Discussion

The present study is the first to analyze hospitalized SLE/RA patients and to compare their relative risk for the occurrence of HF and MI, as well as to evaluate the role of sarcopenia in this patient population. The prevalence of HF and MI in the whole study population was 16.4 and 1.6%, respectively. The overall prevalence of sarcopenia in SLE/RA patients was relatively low (0.1%). These results suggest that sarcopenia may not be associated with increased risk for HF or MI among patients with these autoimmune diseases. Logistic regression analysis showed that RA patients were significantly less likely to have cardiovascular events such as HF or MI than SLE patients after adjusting for relevant confounders.

The increased risk of CVDs, including HF and MI, among SLE patients has been recognized consistently during the past two decades (7–10, 21–23). Specifically, SLE has been reported to be associated with significant alterations in cardiac structure and function (8). Risk of HF is reported to be increased by various SLE manifestations and therapies together with the conventional risk factors for atherosclerotic CVD (9). In the US Medicaid population, the incidence of HF among SLE patients was 2.7-fold higher than among those without SLE (10).

RA has also been found to be associated with increased risk for CVD compared with the general population (24). RA patients were reported to have almost twice the risk of HF than individuals without RA (25). A previous population-based study using the same NIS database as used in the present study documented an increased number of hospitalizations with AMI-RA in the US from 2002 to 2016. More recently, RA was also reported to be associated with lower in-hospital mortality, particularly in cases of STEMI (26).

However, few studies in the medical literature have directly compared CVD risk between RA and SLE. A population-based study in Taiwan found that RA and SLE are associated with in-hospital mortality, overall mortality and major adverse cardiac events either after AMI or stroke (27), although differences in risk of developing MI between the two autoimmune diseases was not studied. Another recent database study focusing on younger adult patients < 55 years reported that SLE was associated with higher risk of AMI, whereas RA was associated with lower risk (28). However, that study still did not directly compare the risk of HF or MI between RA and SLE. An earlier single-center, small-sample study assessed lipid profiles in women with RA or SLE, concluding that women with SLE and RA have a distinct CVD risk profile and the contribution of traditional CV risk factors to atherogenesis may be different in these two autoimmune diseases (29). The present study found that RA patients had a significantly lower risk for MI or HF compared to SLE patients. We surmise that the pathophysiology underlying the distinct cardiovascular risk between RA patients vs. SLE patients could be multifactorial and cannot be answered by the present study design. Nevertheless, these preliminary results highlight directions for future research.

Previous studies have reported that sarcopenia was highly prevalent in HF patients, contributing to its poor prognosis, and suggesting that age-related muscle decline may be an important cause of reduced cardiorespiratory fitness in older adult patients with HF (13–15). Nevertheless, the prevalence of sarcopenia in the present study was relatively low (< 0.2%), and it is probable that the discharge code utilized in the present analysis did not capture sarcopenic patients accurately. Therefore, although the present analysis did not show an increased risk for MI or HF in patients with sarcopenia, a firm conclusion cannot be made on the associations between sarcopenia and risk for MI or HF in patients with autoimmune SLE/RA.

### Strengths and Limitations

The major strengths of this study were the use of the large national representative database of the US, which provided good statistical power. In addition, traditional risk factors for MI or HF were considered and carefully adjusted. However, the study still has several limitations. First, the study uses secondary cross-sectional data and retrospective analysis, both of which may limit generalization to other populations or locations outside the US and prevent long-term follow-up of patients. Also, the lack of information regarding disease duration or activity for SLE and RA populations hindered further adjustment in the analyses. Subgroup analyses based on different severity levels, ejection fraction type and stage of HF, or types of MI (i.e., STEMI, NSTEMI) were also not performed. Lastly, although important, medications used and laboratory parameter values were not provided for patients in the NIS database and could not be included in data analysis, which may have shed light on individual differences between SLE/RA patients. Prospective studies are needed to help elucidate such differences.

## Conclusion

The prevalence of HF and MI in patients with SLE or RA in the US is notable. Patients with RA alone are significantly less likely to have HF or MI than those with SLE alone. Whether sarcopenia leads to increased HF or MI remains inconclusive. Further well-designed prospective studies are highly warranted to explain the pathophysiology underlying discrepancies between RA and SLE regarding risk for MI or HF.

## Data Availability Statement

The original contributions presented in the study are included in the article/supplementary material, further inquiries can be directed to the corresponding author/s.

## Author Contributions

C-MC: acquisition of data, analysis and interpretation of data, drafting of the manuscript, and guarantor of integrity of the entire study. J-RL: acquisition of data, drafting of the manuscript, and statistical analysis. T-CF: conception and design, critical revision of the manuscript, and guarantor of integrity of the entire study. All authors read and approved the final manuscript.

## Funding

This work was supported by Taipei Veterans General Hospital, Taipei, Taiwan and also a grant from Chang Gung Memorial Hospital, Keelung, Taiwan (CMRPG2C0402 and CMRPG2F0193).

## Conflict of Interest

The authors declare that the research was conducted in the absence of any commercial or financial relationships that could be construed as a potential conflict of interest.

## Publisher's Note

All claims expressed in this article are solely those of the authors and do not necessarily represent those of their affiliated organizations, or those of the publisher, the editors and the reviewers. Any product that may be evaluated in this article, or claim that may be made by its manufacturer, is not guaranteed or endorsed by the publisher.

## References

[1] Mok CC To CH Ma KM. Changes in body composition after glucocorticoid therapy in patients with systemic lupus erythematosus. Lupus. (2008) 17:1018–22. 10.1177/0961203308093552 18852226

[2] Sokka T Häkkinen A Kautiainen H Maillefert JF Toloza S Mørk Hansen T. Physical inactivity in patients with rheumatoid arthritis: data from twenty-one countries in a cross-sectional, international study. Arthritis Rheum. (2008) 59:42–50. 10.1002/art.23255 18163412

[3] Benatti FB Pedersen BK. Exercise as an anti-inflammatory therapy for rheumatic diseases-myokine regulation. Nat Rev Rheumatol. (2015) 11:86–97. 10.1038/nrrheum.2014.193 25422002

[4] Hanaoka BY Ithurburn MP Rigsbee CA Bridges S. L. Jr. Moellering DR. Chronic inflammation in rheumatoid arthritis and mediators of skeletal muscle pathology and physical impairment: a review. Arthritis Care Res. (2019) 71:173–7. 10.1002/acr.23775 30295435 PMC6353677

[5] Lilleby V Haugen M Mørkrid L Frey Frøslie K Holven KB Førre O. Body composition, lipid and lipoprotein levels in childhood-onset systemic lupus erythematosus. Scand J Rheumatol. (2007) 36:40–7. 10.1080/03009740600907881 17454934

[6] Hak AE Karlson EW Feskanich D Stampfer MJ Costenbader KH. Systemic lupus erythematosus and the risk of cardiovascular disease: results from the nurses' health study. Arthritis Rheum. (2009) 61:1396–402. 10.1002/art.24537 19790130 PMC2909444

[7] Bartels CM Buhr KA Goldberg JW Bell CL Visekruna M Nekkanti S. Mortality and cardiovascular burden of systemic lupus erythematosus in a US population-based cohort. J Rheumatol. (2014) 41:680–7. 10.3899/jrheum.130874 24532834 PMC3975689

[8] Chen J Tang Y Zhu M Xu A. Heart involvement in systemic lupus erythematosus: a systemic review and meta-analysis. Clin Rheumatol. (2016) 35:2437–48. 10.1007/s10067-016-3373-z 27502777

[9] Dhakal BP Kim CH Al-Kindi SG Oliveira GH. Heart failure in systemic lupus erythematosus. Trends Cardiovasc Med. (2018) 28:187–97. 10.1016/j.tcm.2017.08.015 28927572

[10] Chen SK Barbhaiya M Fischer MA Guan H Yoshida K Feldman CH. Heart failure risk in systemic lupus erythematosus compared to diabetes mellitus and general medicaid patients. Semin Arthritis Rheum. (2019) 49:389–95. 10.1016/j.semarthrit.2019.06.005 31280938 PMC6918835

[11] Chung WS Lin CL Peng CL Chen YF Lu CC Sung FC. Rheumatoid arthritis and risk of acute myocardial infarction–a nationwide retrospective cohort study. Int J Cardiol. (2013) 168:4750–4. 10.1016/j.ijcard.2013.07.233 23938220

[12] Myasoedova E Davis JM Roger VL Achenbach SJ Crowson CS. Improved incidence of cardiovascular disease in patients with incident rheumatoid arthritis in the 2000s: a population-based cohort study. J Rheumatol. (2021) 48:1379–87. 10.3899/jrheum.200842 33589553 PMC8364571

[13] Carbone S Lavie CJ Arena R. Obesity and heart failure: focus on the obesity paradox. Mayo Clin Proc. (2017) 92:266–79. 10.1016/j.mayocp.2016.11.001 28109619

[14] Curcio F Testa G Liguori I Papillo M Flocco V Panicara V. Sarcopenia and heart failure. Nutrients. (2020) 12:211. 10.3390/nu12010211 31947528 PMC7019352

[15] Lena A Anker MS Springer J. Muscle wasting and sarcopenia in heart failure-the current state of science. Int J Mol Sci. (2020) 21:6549. 10.3390/ijms21186549 32911600 PMC7555939

[16] Santos MJ Vinagre F Canas da Silva J Gil V Fonseca JE. Body composition phenotypes in systemic lupus erythematosus and rheumatoid arthritis: a comparative study of Caucasian female patients. Clin Exp Rheumatol. (2011) 29:470–6. Available online at: https://www.clinexprheumatol.org/abstract.asp?a=4190 21640047

[17] Brance ML Di Gregorio S Pons-Estel BA Quagliato NJ Jorfen M Berbotto G. Prevalence of sarcopenia and whole-body composition in rheumatoid arthritis. J Clin Rheumatol. (2021) 27:S153–60. 10.1097/RHU.0000000000001549 32897991

[18] Ngeuleu A Allali F Medrare L Madhi A Rkain H Hajjaj-Hassouni N. Sarcopenia in rheumatoid arthritis: prevalence, influence of disease activity and associated factors. Rheumatol Int. (2017) 37:1015–20. 10.1007/s00296-017-3665-x 28258473

[19] HCUP. Introduction to the Nationwide Inpatient Sample (NIS). Rockville, MD: Agency for Healthcare Research and Quality (2008).

[20] Lin MH Chiu SY Chang PH Lai YL Chen PC Ho WC. Hyperlipidemia and statins use for the risk of new diagnosed sarcopenia in patients with chronic kidney: a population-based study. Int J Environ Res Public Health. (2020) 17:1494. 10.3390/ijerph17051494 32110901 PMC7084510

[21] Ward MM. Premature morbidity from cardiovascular and cerebrovascular diseases in women with systemic lupus erythematosus. Arthritis Rheum. (1999) 42:338–46. 10.1002/1529-0131(199902)42:2andlt;338::AID-ANR17andgt;3.0.CO;2-U 10025929

[22] Pons-Estel GJ González LA Zhang J Burgos PI Reveille JD Vilá LM. Predictors of cardiovascular damage in patients with systemic lupus erythematosus: data from LUMINA (LXVIII), a multiethnic US cohort. Rheumatology. (2009) 48:817–22. 10.1093/rheumatology/kep102 19454606 PMC2722811

[23] Kim CH Al-Kindi SG Jandali B Askari AD Zacharias M Oliveira GH. Incidence and risk of heart failure in systemic lupus erythematosus. Heart. (2017) 103:227–33. 10.1136/heartjnl-2016-309561 27613169

[24] Fragoulis GE Panayotidis I Nikiphorou E. Cardiovascular risk in rheumatoid arthritis and mechanistic links: from pathophysiology to treatment. Curr Vasc Pharmacol. (2020) 18:431–46. 10.2174/1570161117666190619143842 31258091

[25] Park E Griffin J Bathon JM. Myocardial dysfunction and heart failure in rheumatoid arthritis. Arthritis Rheumatol. (2022) 74:184–99. 10.1002/art.41979 34523821 PMC8795468

[26] Elbadawi A Ahmed HMA Elgendy IY Omer MA Ogunbayo GO Abohamad S. Outcomes of acute myocardial infarction in patients with rheumatoid arthritis. Am J Med. (2020) 133:1168–79.e4. 10.1016/j.amjmed.2020.02.039 32278845

[27] Lai CH Hsieh CY Barnado A Huang LC Chen SC Tsai LM. Outcomes of acute cardiovascular events in rheumatoid arthritis and systemic lupus erythematosus: a population-based study. Rheumatology. (2020) 59:1355–63. 10.1093/rheumatology/kez456 31600392 PMC7849999

[28] Krittanawong C Liu Y Mahtta D Narasimhan B Wang Z Jneid H. Non-traditional risk factors and the risk of myocardial infarction in the young in the US population-based cohort. Int J Cardiol Heart Vasc. (2020) 30:100634. 10.1016/j.ijcha.2020.100634 32995474 PMC7516292

[29] Santos MJ Vinagre F Silva JJ Gil V Fonseca JE. Cardiovascular risk profile in systemic lupus erythematosus and rheumatoid arthritis: a comparative study of female patients. Acta Reumatol Port. (2010) 35:325–32. Available online at: https://www.arprheumatology.com/oldsite/conteudo/pdfs/08._AO_-_FR_CV_LES_ARP2010.67AO.pdf 20975635

